# Review of interruptions in a pediatric subspecialty outpatient clinic

**DOI:** 10.1371/journal.pone.0254528

**Published:** 2021-07-29

**Authors:** Tyler Lee, Hinette Rosario, Elizabeth Cifuentes, Jiawei Cui, Emery C. Lin, Victoria A. Miller, Henry C. Lin

**Affiliations:** 1 Philadelphia College of Osteopathic Medicine, Philadelphia, PA, Division of Gastroenterology, Hepatology, and Nutrition, Children’s Hospital of Philadelphia, Philadelphia, Pennsylvania, United States of America; 2 Division of Gastroenterology, Hepatology, and Nutrition, Children’s Hospital of Philadelphia, Philadelphia, Pennsylvania, United States of America; 3 Department of Medicine, Oregon Health & Science University, Portland, Oregon, United States of America; 4 Division of Adolescent Medicine, Children’s Hospital of Philadelphia, Philadelphia, PA, Perelman School of Medicine, University of Pennsylvania, Philadelphia, Pennsylvania, United States of America; 5 Division of Pediatric Gastroenterology, Doernbecher Children’s Hospital, Department of Pediatrics, Oregon Health & Science University, Portland, Oregon, United States of America; University of Adelaide, AUSTRALIA

## Abstract

**Introduction:**

The objective of this study was to describe interruptions in the pediatric ambulatory setting and to assess their impact on perceived physician communication, patient satisfaction and recall of provided physician instructions.

**Methods:**

An observational study was performed at the Children’s Hospital of Philadelphia, Pediatric Gastroenterology clinic. Participation consisted of video recording the clinic visit and the caregiver completed post-visit surveys on communication and satisfaction. Video recordings were coded for interruptions, which were divided into 3 main categories: Visit Associated, Pediatric Associated, and Unanticipated. An interruption rate was calculated and correlated with the following outcome variables to assess the impact of interruptions: caregiver satisfaction, caregiver perception on the quality of physician communication, and caregiver instruction recall.

**Results:**

There were 675 interruptions noted in the 81 clinic visits, with an average of 7.96 (σ = 7.68) interruptions per visit. Six visits had no interruptions. The Patient was the most frequent interrupter. Significantly higher interruption rates occurred in clinic visits with younger patients (<7 years old) with most of the interruptions being Pediatric Associated interruptions. There was minimal correlation between the clinic visit interruption rate and caregiver satisfaction with the communication, caregiver perception of quality of communication, or caregiver instruction recall rate.

**Conclusion:**

The effect of interruptions on the pediatric visit remains unclear. Interruptions may be part of the communication process to ensure alignment of the patient’s agenda. Additional studies are needed to help determine the impact of interruptions and guide medical education on patient communication.

## Introduction

Over the past 50 years, there have been tremendous reform efforts aimed at shifting the doctor-patient interaction from one of physician authoritarianism on the part of the physician to one that allows for harmonious collaboration between physician and patient. Communication is vital to this relationship and there has been an increasing emphasis placed on optimizing physician communication during clinical encounters in order to deliver exemplary patient-centered care [1–4]. In pediatrics, the 3 key elements to physician-parent-child communication include informativeness, interpersonal sensitivity, and partnership building [5, 6]. Interruptions may negatively affect these key communication elements with regards to transmittal of patient information or development of patient rapport [7–10].

An interruption is any act that physically disrupts the flow of speech or inhibits further development of a topic. Beckman and Frankel (1984) identified four types of verbal interruptions can disrupt the flow of conversation and potentially affect the process of patient agenda setting [11, 12]. In addition to these verbal interruptions, there are many other external distractors in the ambulatory clinic setting including phone calls and pagers. Furthermore, in pediatrics, the patient and sibling can also be a source or distractions and potential interruptions.

Byrne and Long’s (1976) findings that patients are often interrupted while presenting their problems, highlighted the role of communication and drew attention to the impact of interruptions during the clinical visit [13]. Numerous studies have reported that interruptions are abundant during adult outpatient visits with up to 75% of patients being interrupted by their physicians before finishing speaking. This interruption reduced the average patient speaking time during the initial problem presentation period from 73–150 seconds to 12–23 seconds [10, 11, 14]. In particular, Beckman and Frankel (1984) observed a mean time to initial interruption of 18 seconds, well before the patient completed their initial statement [11].

Part of the impetus behind provider driven interruptions may be due to decreasing facetime with patients as a 2017 survey, pediatricians most frequently reporting having only 13–16 minutes with the patient [15]. These interruptions may result in the physician controlling the conversation [8, 16] and can potentially lead to a scenario in which the patient is not able to fully share concerns. In addition, there is a gender disparity with interruptions with some studies suggesting that female physicians were more likely to be interrupted by their patients than male physicians [17].

The impact of interruptions on the clinic visit remains unclear. Some studies report a predominantly disruptive impact of physician interruptions, especially when used as a tactic to gain control of the conversation [18–21]. Interruptions can increase cognitive burden which can lead to loss of concentration, delays in work, and even unintentional errors [22, 23]. Not all interruptions are negative as physician initiated interruptions can play a role in establishing the physician’s expertise [24]. Thoughtful provider-initiated interruptions can help with patient care coordination [21, 25–27] and can also be an expression of support and collaboration [28]. These studies on the impact of interruptions in the medical setting have primarily been focused on adults and there is limited literature on the type and role of interruptions in pediatrics.

The objective of this study was to describe interruptions in the pediatric ambulatory setting and to assess their impact on perceived physician communication, patient satisfaction and recall of provided physician instructions.

## Methods

Ethics approval and consent to participate was reviewed and approved by the Children’s Hospital of Philadelphia’s Institutional Review Board. Written informed consent was obtained for participation in the study.

An observational study was performed at the Children’s Hospital of Philadelphia, Pediatric Gastroenterology (GI) outpatient clinic from February 2016 to August 2016. Participants in study were the patient and caregiver, and were drawn from a convenience sample of patients seen in the GI clinic. The study consisted of video recording the visit and the caregiver completed a post-visit survey on communication and satisfaction. Consent was also obtained from the physicians. A research assistant was present in the room for the entire visit. Subjects had the option to stop the video recording any time during the visit. The nature of video recording a visit to study patient communication lends itself to response bias by both the provider and patient. The research assistant was placed in a corner of the room to be as unobtrusive as possible. Participants were also assured that all survey responses and study findings were deidentified.

Outcome variables to assess the impact of interruptions included caregiver satisfaction, caregiver perception on the quality of physician communication, and caregiver instruction recall. Caregiver communication satisfaction was assessed with a previously validated version the Patient Satisfaction Questionnaire 18 (Modified PSQ-18) [29, 30]. The Kalamazoo Essential Elements Communication Checklist (KEECC), a validated measure of medical communication, was used to assess caregiver perception of the quality of physician communication [31, 32]. Both of these surveys were scored on a 5-point Likert scale allowing for a quantitative measure of satisfaction (Modified PSQ-18) and communication quality (KEECC). Within seven days of the clinic visit, caregivers were called to assess their retention of the written physician instructions which were provided and verbally reviewed at the conclusion of the clinic visit. Instruction recall rate was calculated based on the total number of the written instructions provided.

Video recordings of each visit were divided into 4 separate sections: Introduction, History Gathering, Exam, and Summary. Each video was coded for interruptions by 3 different research assistants with differences in coding resolved by discussion among the research assistants. Interruptions were categorized by interrupter and by type of interruption. Interrupters include the physician, patient, caregiver, sibling, healthcare worker, research assistant, and other. Types of interruptions were divided into 3 categories: Visit Associated, Pediatric Associated, and Unanticipated. Visit associated verbal interruptions are those that occur with the clinical dialogue and have previously been characterized by various studies to include: expressing agreement (A), asking for clarification (C), and other questions (Q). Pediatric Associated interruptions are those that are unique to the pediatric setting and include: interaction by caregiver with patient or sibling (I), playing (P), and talking, crying or yelling by the patient or sibling (Y). Unanticipated interruptions include: telephone or pager distraction (T), and entering or exiting the room (E).

Descriptive statistics were used to summarize findings from the video recording analysis and presented as mean and standard deviation. Descriptive data includes total interruptions, time to first interruption, type of interruptions and person interrupting. To compare frequency of interruptions, an interruption rate of ‘interruptions per 5 minutes’ was calculated for each visit. One-way ANOVA tests, as well as two-sample t-tests, were used to compare number of interruptions and interruption rates among subject and clinic demographics.

Ethics approval was obtained by the Children’s Hospital of Philadelphia’s Institutional Review Board (IRB). A secure, password protected server was used to store the video recordings and the initial recording was destroyed to protect patient confidentiality.

## Results

171 subjects were approached and 81 participated in the study. The primary reason for declining participation was due to video recording the visit. All 81 subjects completed the video recording and follow-up surveys. The subjects were seen by 11 different physicians: 3 male and 8 female who ranged between 1–15 years of clinical experience. There were 35 new patient visits and 46 follow-up visits. Patients ranged in age from infancy to 17 years old, with 22.2% infants or toddlers, 16.1% preschoolers (3–4.9 years), 43.2% grade-schoolers (5–12 years), and 18.5% teenagers (13–18 years). A single caregiver was present for the majority of visits (71.6%). Siblings were present in 15% of the visits (n = 12).

The average clinic visit time was 26.13 minutes with the longest time spent during the History Gathering section (Table 1). New patient visits took significantly more time than follow-up visits (32.85 min vs 21.03 min, t(64) = 5.31, p < 0.01) with the History Gathering and Summary sections being significantly longer for new patient visits (History Gathering: 15.82 min vs 9.66 min, t(49) = 4.07, p < 0.01; Summary: 12.41 min vs 8.48 min, t(67) = 3.75, p < 0.01).

**Table 1:** Time distribution and interruption rate distribution.

	Introduction	History	Exam	Summary	Overall
**Average Time (min)**	0.78 ± 0.65	12.32 ± 6.95	2.84 ± 2.72	10.17 ± 4.95	26.13 ± 11.25
	(2.99%)	(47.15%)	(10.91%)	(38.96%)	
New Visit	0.74 ± 0.56	15.82 ± 8.10	3.65 ± 2.75	12.41 ± 4.98	32.85 ± 10.74
	(2.25%)	(48.16%)	(11.11%)	(37.78%)	
Follow-Up	0.80 ± 0.71	9.65 ± 4.41	2.23 ± 2.56	8.47 ± 4.24	21.03 ± 8.73
	(3.80%)	(45.89%)	(10.60%)	(40.28%)	
**Average Number of Interruptions**	0.22 ± 0.50	4.98 ± 5.15	0.35 ± 0.78	2.82 ± 4.11	8.39 ± 8.00
	(2.62%)	(59.36%)	(4.17%)	(33.61%)	
New Visit	0.22 ± 0.55	5.77 ± 5.48	0.45 ± 0.95	3.71 ± 5.29	10.17 ± 8.87
	(2.16%)	(56.74%)	(4.24%)	(36.48%)	
Follow-Up	0.21 ± 0.47	4.39 ± 4.86	0.28 ± 0.62	2.15 ± 2.79	7.04 ± 7.08
	(2.98%)	(62.36%)	(3.98%)	(30.54%)	
**Average Interruption Rate (int/5min)**	1.42	2.02	0.63	1.39	1.61
New Visit	1.53	1.82	0.63	1.50	1.55
Follow-Up	1.35	2.27	0.63	1.27	1.67

There were 675 interruptions during the 81 clinic visits. Six visits (7.4%) had no interruptions throughout the entire visit. There was an average of 7.96 (σ = 7.68) interruptions per visit with an average interruption rate of 1.81 interruptions per 5 minutes (int/5min). The History Gathering section had the highest interruption rate of 2.02 int/5min (Table 1). There were no significant differences in interruption rate between the different sections of the visit. There was no significant difference in overall interruption rate between new visits and follow-up visits.

The median time to first interruption instance was 105 seconds. The majority (71.6%) of the first interruption instances occurred during the History gathering section of the visit while 18.5% of the first interruptions occurred during the Introduction. First interruptions were most often initiated by the physician (41.4%) followed by the patient (28.0%), caregiver (20.0%), and other (10.6%).

Pediatric Associated interruptions were the most common category of interruptions (55.3%) followed by Visited Associated, and then Unanticipated (Table 2). The top three types of interruptions were talking, crying or yelling by the patient or sibling (Y), asking for clarification, and telephone or pager distraction. Clarification interruptions were primarily initiated by the physician (57%) and telephone interruptions primarily occurred with caregivers (60.8%). New patient visits had significantly more Clarification type interruptions as compared to follow up visits (2.91 vs 1.52, t(57) = 2.50, p = 0.015).

**Table 2:** Clinic visit distribution of interruption types.

Interruption Type (Count)		Introduction	History	Exam	Summary	Subtotal And Percentage	Total
Visit Associated	Clarification (C)	1	134	1	36	172 (89.6%)	192
	Question (Q)	0	1	0	14	15	
						(7.8%)	(28.4%)
	Agreement (A)	0	3	0	2	5	
						(2.6%)	
	**Subtotal**	**1**	**138**	**1**	**52**		
Pediatric Associated	Yell/Cry/Talk (Y)	6	139	15	95	255 (68.4%)	373
	Interaction (I)	2	27	0	21	50	
						(13.4%)	(55.3%)
	Playing (P)	1	38	1	28	68	
						(18.2%)	
	**Subtotal**	**9**	**204**	**16**	**144**		
Unanticipated	Technological (T)	4	49	8	19	80	110
						(72.7%)	
	Exit (E)	2	11	4	13	30	(16.3%)
						(27.3%)	
	**Subtotal**	**6**	**60**	**12**	**32**		
**Total**		**16**	**402**	**29**	**228**		**675**

The Patient was the most frequent interrupter during the clinic visit accounting for 50.8% of the total interruptions, followed by the physician, and then the caregiver (Table 3). Both the physician and caregiver interrupted most frequently during the History Gathering section. Physicians interrupted significantly more during the History Gathering than the other portions of the visit combined (102 interruptions versus 39 total interruptions during other sections of the visit, t(114) = 3.81, p = 0.0002). There was no gender difference in who initiated interruptions.

**Table 3:** Clinic distribution of interrupters.

Interrupter (Count, Percentage)	Introduction		History		Exam		Summary		Total	
Physician	7	43.8%	102	25.3%	4	13.8%	28	12.3%	141	20.9%
Patient	7	43.8%	192	47.8%	14	48.3%	130	57.0%	343	50.8%
Caregiver	1	6.2%	78	19.4%	4	13.8%	46	20.2%	129	19.1%
Patient Sibling	1	6.2%	16	4.0%	4	13.8%	15	6.6%	36	5.3%
Other	0	0%	14	3.5%	3	10.3%	9	3.9%	26	3.9%
**Total**	**16**		**402**		**29**		**228**		**675**	

**Fig 1:**
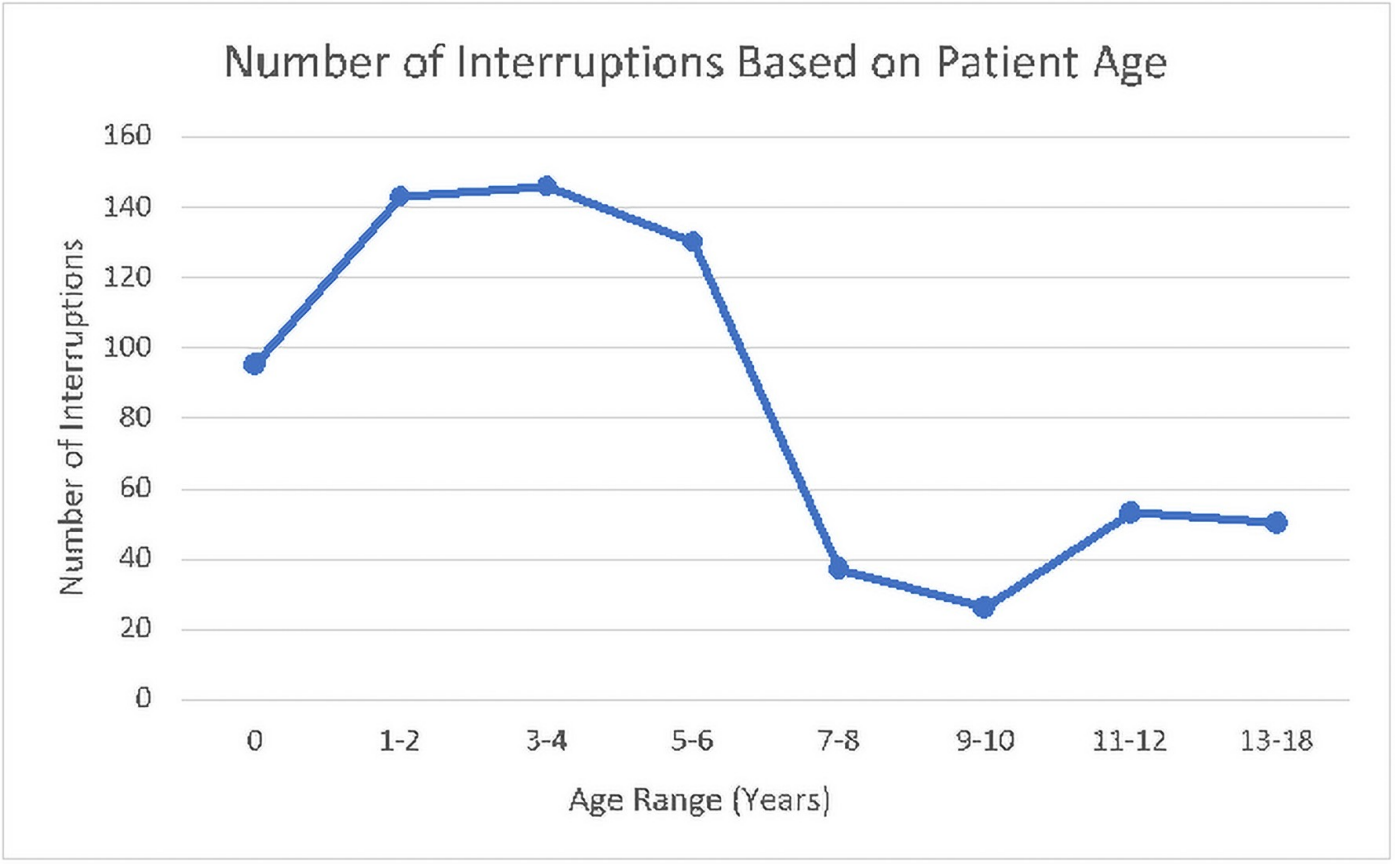
Number of interruptions based on patient age.

Clinic interruption rates varied by patient age (Fig 1). Significantly higher interruption rates occurred in clinic visits with younger patients (<7 years old) compared to visits with older patients (7–18 years) (3.25 int/5min vs 0.81 int/5min, t(39) = 4.08, p < 0.01). Most of these interruptions in visits with patients <7 years old were Pediatric Associated interruptions and primarily initiated by the patient. Younger patients had significantly more Y-type interruptions (5.42 per visit vs 1.13, t(63) = 4.01, p = 0.0001). Interruption rates by the Patient, Caregiver, and Physician were all higher in visits with younger patients: patient (2.05 int/5min vs 0.63 int/5min, t(43) = 3.12, p < 0.01), caregiver (0.63 int/5min vs 0.40 int/5min, t(36) = 2.18, p = 0.03), and physician (0.69 int/5min vs 0.28 int/5min, t(38) = 4.70, p < 0.01).

The impact of interruptions on the clinic visit was assessed by the following outcome data: caregiver satisfaction with communication (Modified PSQ-18), caregiver perception of quality of communication (KEECC), and caregiver instruction recall rate. The average caregiver satisfaction with communication was 4.73 out of 5 (σ = 0.51). The average caregiver perception of communication quality was 4.67 out of 5 (σ = 0.56). The average caregiver instruction recall rate was 72% (σ = 0.31). There was minimal correlation between the clinic visit interruption rate and each of these outcome metrics (Table 4). There was also no correlation between time to first interruption and patient satisfaction.

**Table 4:** Correlation between visit interruption rate and different outcome metrics.

		Communication Satisfaction*	Communication Quality^$^	Instruction Recall^+^
**Interruption Rate**	**Overall**	-0.092	-0.023	0.149
	**- Visit**	0.091	0.081	0.183
	**- Pediatric**	-0.128	-0.044	0.068
	**- Unanticipated**	-0.042	-0.003	0.133

* Communication satisfaction measured via the Modified PSQ-18

^$^ Communication quality measured via the Kalamazoo Essential Elements Communication Checklist

^+^ Instruction recall rate was calculated by the number of instructions remembered based on the total number of the written instructions provided

## Discussion

While many studies have sought to understand the complex nature of interruptions and their roles, there are limited studies describing interruptions in the pediatric ambulatory setting or assessing the impact of these interruptions. In this study, we focused on the dynamic interaction among the patient-caregiver-physician triad and describe interruptions by timing, type, and interrupter that occurred pediatric gastroenterology ambulatory clinic visits at the Children’s Hospital of Philadelphia.

A common metric for studies on interruptions is time to initial interruption. In adult studies, the average time to initial interruption has been reported between 12–18 seconds [10, 11]. Uninterrupted, the median time for adult patients to state their primary medical concern has been reported at only 6 seconds [33]. Rhoades et al (2001) reported that the while the time to initial interruption was 12 seconds, the patient was interrupted during their initial statement 25% of the time [10]. Interruptions thus serve as a medium for one to gain control of the medical conversation, and for physicians, possibly as a way to expeditiously direct the conversation given the time constraints from decreased facetime with patients [15]. For example, Marvel et al (1999) reported that physicians redirected a patient’s initial statement after an average of 23.1 seconds but patients who were uninterrupted took an average of 6 seconds more to finish their initial statement [14]. While interruption may help direct the conversation, the patient may not take long to complete their initial statement and allowing patients to complete their initial thoughts may improve the quality of communication for the remainder of the visit.

In our study, the average time to interruption was 105 seconds, which is much longer than the previously reported times to interruptions. Theoretically, allowing the patient or caregiver to speak uninterrupted initially can help with patient agenda setting and gathering of more information. With a comparatively longer time to initial interruption, it is conceivable that our patients and caregivers had ample time to express their concerns, which may account for the generally positive caregiver satisfaction with communication and perception of the quality of communication. It is unclear if this observation of allowing the caregiver more uninterrupted time to speak is unique to our study or to pediatrics or to external factors. One possible reason for longer uninterrupted speaking time by the patient or caregiver could be attributed to the focus on communication as part of the medical curriculum as interpersonal skills and communication is one of the six Accreditation Council for Graduate Medical Education (ACGME) core competencies [34, 35].

Previous studies are inconclusive in whether the physician or adult patient interrupts more frequently [9, 17, 36, 37]. Realini et al (1995) reported that while the patient and physician were similarly likely to gain control over the conversation, the person initiating the interruption more often gained control of the conversation [16]. While physicians initiated the majority of the initial interruptions, the patient was the most common interrupter overall and we did not assess who gained control of the conversation following an interruption.

The observation that the patient was the most frequent interrupter is not surprising as in pediatrics, younger children may be unaware of the social situation or norms. In particular, we noted that in clinic visits with children younger than 7 years old, there were more interruptions in general, with the patient, caregiver, and physician all having higher interruptions rates. Part of this increase in interruptions could be attributed to the child interrupting or requiring interactions to allow the caregiver and physician to speak. It is also possible that caregivers of younger patients may require a different communication approach as younger children are less likely to clearly express their medical condition, requiring caregivers to engage the patient and ask more questions as they try to comprehend the child’s complaints.

Also unique to pediatrics are interruptions that do not target the typical medical communication process. We termed these events as Pediatric Associated interruptions, which includes yelling or crying, playing or making noise, or an interaction with the child. Not surprisingly, Pediatric Associated interruptions were the most common type of interruptions noted. While these interruptions may still affect the flow of conversation, they are distinct in that they are not designed to gain control of the medical conversation. Likewise, unanticipated interruptions can affect the flow of conversation. With the majority of Americans owning a cell phone, technological interruptions are no longer limited to the physician being paged [38]. Incoming texts or phone calls can temporarily distract from the medical conversation and in our study, we observed the physician, caregiver, and patient all initiating technological interruptions. As the profession continues to work to improve communication quality, it will be important to determine the effect of pediatric associated and technology-based interruptions on quality of care.

To assess the impact of interruptions, we selected three outcomes that could potentially influenced by interruptions: caregiver satisfaction with communication, caregiver perception of quality of communication, and caregiver instruction recall rate. We did not observe any correlation between the overall clinic interruption rate and any of these metrics. It is possible that this lack of correlation could be due to the fact that the definition of an interruption as perceived by the caregiver is different than what our study defined as an interruption. For example, a pediatric associated interruption may not be perceived by caregivers as an interruption that affects communication satisfaction. It is also possible that pediatric providers are accustomed to dealing with these types of interruptions as interruptions by the child are inherent to pediatrics. Perhaps, both the provider and caregiver have adapted their communication and processing style to account for such interruptions. To assess for this possibility, sub-group correlations were performed with Visit Associated, Pediatric Associated, and Unanticipated interruptions, but there was minimal correlation between the subgroup interruption rates and the outcome metrics. Unanticipated interruptions include technological disruptions, which accounted for 11.8% of the total interruptions noted (80/675). There was minimal correlation between technological interruptions and patient satisfaction, perceived quality of communication, or instruction recall. The lack of correlation between interruption rate and instruction recall could be due to minimal interruptions occurring during the instruction review process, with most interruptions during instruction review focused on clarification. The minimal negative correlation of interruption frequency with communication quality could be a reflection that caregivers do not view provider-initiated clarification, agreement, or question interruptions as a negative verbal tactic but rather as a part of the communication process designed to elicit necessary medical information. Furthermore, caregiver-initiated interruptions may allow the caregiver to express unmet concerns. In these instances, interruptions could aid in the quality of communication or overall satisfaction by helping with building rapport and cooperation [37], but a positive correlation between interruption rate and communication quality was not observed either. To better understand the effect of interruptions, in future studies, caregivers should be asked if they felt interrupted.

One of the main limitations to this study is the Hawthorne effect, as patient, caregiver, and physician may have acted differently knowing that they were being video recorded. Likewise, having a research coordinator in the room may have also disrupted the typical clinic setting. However, unique to pediatrics is that the physician is usually being observed by a third party (caregiver) especially when the patient is old enough to provide the medical history. Another consideration would be if these measures of communication quality and satisfaction do not accurately reflect the caregiver’s perceptions as while both the KEECC and Modified PSQ-18 are validated measures, they have not been used in the pediatric setting. Lastly, it is also possible that what was defined as an interruption was not perceived as an interruption by the caregiver or physician, which may affect the data on assessing for impact of interruptions.

## Conclusion

In our description of interruptions in the pediatric outpatient setting, interruptions occurred more frequently in clinic visits with younger patients with the patient being the most frequent interrupter. The effect of these interruptions on the clinical visit remains unknown. Further studies are needed and could include assessing caregiver and physician perception on interruptions to determine if certain types of interruptions are beneficial or disruptive.

Overall, despite time demands on physicians, the goal remains to communicate effectively to acknowledge and address the patient’s concerns. Interruptions may be part of the communication process to allow both physician and caregiver to ensure alignment of the patient’s agenda. As this study was in a subspecialty clinic setting, replication in a primary care setting could help determine the impact of interruptions and guide future medical education on patient communication.

## Supporting information

S1 File (XLSX)

## References

[1] Chin JJ, Doctor-patient relationship: from medical paternalism to enhanced autonomy. Singapore Med J, 2002. 43(3): p. 152–5. 12005343

[2] Teutsch C, Patient-doctor communication. Med Clin North Am, 2003. 87(5): p. 1115–45. doi: 10.1016/s0025-7125(03)00066-x 14621334

[3] Kaplan SH, Greenfield S, Gandek B, Rogers W.H., Ware J.E, Characteristics of physicians with participatory decision-making styles. Ann Intern Med, 1996. 124(5): p. 497–504. doi: 10.7326/0003-4819-124-5-199603010-00007 8602709

[4] Hall JA, Horgan T, Stein T, Roter D, Liking in the physician--patient relationship. Patient Educ Couns, 2002. 48(1): p. 69–77. doi: 10.1016/s0738-3991(02)00071-x 12220752

[5] Street RL, Physicians’ communication and parents’ evaluations of pediatric consultations. Med Care, 1991. 29(11): p. 1146–52. doi: 10.1097/00005650-199111000-00006 1943273

[6] Mauksch LB, Dugdale DC, Dodson S, Epstein R, Relationship, communication, and efficiency in the medical encounter: creating a clinical model from a literature review. Arch Intern Med, 2008. 168(13): p. 1387–95. doi: 10.1001/archinte.168.13.1387 18625918

[7] Schegloff E, Jefferson G, Sacks H., The Preference for Self-Correction in the Organization of Repair in Conversation. Language, 1977. 53: p. 361–382.

[8] Frankel RM, From sentence to sequence: understanding the medical encounter through microinteractional analysis. Discourse Process, 1984. 7: p. 135–170.

[9] Irish JT, Hall JA, Interruptive patterns in medical visits: the effects of role, status and gender. Soc Sci Med, 1995. 41(6): p. 873–81. doi: 10.1016/0277-9536(94)00399-e 8571159

[10] Rhoades DR, McFarland KF, Finch WH, Johnson AO, Speaking and interruptions during primary care office visits. Fam Med, 2001. 33(7): p. 528–32. 11456245

[11] Beckman HB, Rankel RM, The effect of physician behavior on the collection of data. Ann Intern Med, 1984. 101(5): p. 692–6. doi: 10.7326/0003-4819-101-5-692 6486600

[12] Mauksch LB, Questioning a Taboo: Physicians’ Interruptions During Interactions With Patients. JAMA, 2017. 317(10): p. 1021–1022. doi: 10.1001/jama.2016.16068 28291896

[13] Byrne PS, Long BE, *Doctors talking to patients*: *a study of the verbal behaviour of general practitioners consulting in their surgeries*. 1976, London: HMSO.

[14] Marvel MK, Epstein RM, Flowers K, Beckman HB, Soliciting the patient’s agenda: have we improved? JAMA, 1999. 281(3): p. 283–7. doi: 10.1001/jama.281.3.283 9918487

[15] Medscape Physicain Compensation Report 2017, 2017, https://www.medscape.com/slideshow/compensation-2017-overview-6008547. Accessed 5 December 2020.

[16] Realini T, Kalet A, Sparling J, Interruption in the medical interaction. Arch Fam Med, 1995. 4(12): p. 1028–33. doi: 10.1001/archfami.4.12.1028 7496551

[17] West C, When the doctor is a "lady": Power, status and gender in physician-patient encounters. Symbolic Interaction, 1984. 7: p. 87–106.

[18] Waitzkin H, Doctor-patient communication. Clinical implications of social scientific research. JAMA, 1984. 252(17): p. 2441–6. 6481931 10.1001/jama.252.17.2441

[19] Mishra S, Waitzkin H, Physician-patient communication. West J Med, 1987. 147(3): p. 328. 18750322 PMC1025872

[20] Koong AY, Koot D, Eng SK, Purani A, Yusoff A, Goh CC, et al., When the phone rings—factors influencing its impact on the experience of patients and healthcare workers during primary care consultation: a qualitative study. BMC Fam Pract, 2015. 16: p. 114. doi: 10.1186/s12875-015-0330-x 26330170 PMC4557219

[21] Goldberg JA, Interrupting the discourse on interruptions: An Analysis in Terms of Relationally Neutral, Power-and Rapport-Oriented Acts. Journal of Pragmatics, 1990. 14: p. 883–903.

[22] Blocker RC, Heaton HA, Forsyth K, Hawthorne HJ, El-Sherif N, Bellolio M, et al., Physician, Interrupted: Workflow Interruptions and Patient Care in the Emergency Department. J Emerg Med, 2017. 53(6): p. 798–804. doi: 10.1016/j.jemermed.2017.08.067 29079489

[23] McGillis Hall L, Pedersen C, Hubley P, Ptack E, Hemingway A, Watson C, et al., Interruptions and pediatric patient safety. J Pediatr Nurs, 2010. 25(3): p. 167–75. doi: 10.1016/j.pedn.2008.09.005 20430277

[24] Mischler EG, *The discourse of medicine*: *The dialects of medical interviews*. 1984, Norwood, NJ: Ablex.

[25] Schneider A, Wehler M, Weigl M, Provider interruptions and patient perceptions of care: an observational study in the emergency department. BMJ Qual Saf, 2019. 28(4): p. 296–304. doi: 10.1136/bmjqs-2018-007811 30337495

[26] Rivera-Rodriguez AJ, Karsh BT, Interruptions and distractions in healthcare: review and reappraisal. Qual Saf Health Care, 2010. 19(4): p. 304–12. doi: 10.1136/qshc.2009.033282 20378621 PMC3007093

[27] Grundgeiger T, Dekker S, Sanderson P, Brecknell B, Liu D, Aitken LM, Obstacles to research on the effects of interruptions in healthcare. BMJ Qual Saf, 2016. 25(6): p. 392–5. doi: 10.1136/bmjqs-2015-004083 26658346

[28] Larsson US, Saljo R, Aronsson K, Patient-doctor communication on smoking and drinking: lifestyle in medical consultations. Soc Sci Med, 1987. 25(10): p. 1129–37. doi: 10.1016/0277-9536(87)90354-6 3686078

[29] Marshall GN, Hays RD, *The Patient Satisfaction Questionnaire Short Form (PSQ-18)*. 1994, RAND Corporation: Santa Monica, CA.

[30] Oda Y, Onishi H, Sakemi T, Fujimoto K, Koizumi S, Improvement in medical students’ communication and interpersonal skills as evaluated by patient satisfaction questionnaire after curriculum reform. J Clin Biochem Nutr, 2014. 55(1): p. 72–7. doi: 10.3164/jcbn.14-29 25120283 PMC4078071

[31] Calhoun AW, Rider EA, Meyer EC, Lamiani G, Truog RD, Assessment of communication skills and self-appraisal in the simulated environment: feasibility of multirater feedback with gap analysis. Simul Healthc, 2009. 4(1): p. 22–9. doi: 10.1097/SIH.0b013e318184377a 19212247

[32] Makoul G, Essential elements of communication in medical encounters: the Kalamazoo consensus statement. Acad Med, 2001. 76(4): p. 390–3. doi: 10.1097/00001888-200104000-00021 11299158

[33] Singh Ospina N, Phillips KA, Rodriguez-Gutierrez R, Castaneda-Guarderas A, Biofriddo MR, Branda ME, et al, Eliciting the Patient’s Agenda- Secondary Analysis of Recorded Clinical Encounters. J Gen Intern Med, 2019. 34(1): p. 36–40. doi: 10.1007/s11606-018-4540-5 29968051 PMC6318197

[34] Benson BJ, Domain of competence: Interpersonal and communication skills. Acad Pediatr, 2014. 14(2 Suppl): p. S55–65. doi: 10.1016/j.acap.2013.11.016 24602649

[35] Duffy FD, Fordon GH, Whelan G, Cole-Kelly K, Frankel R, Buffone N, et al., Assessing competence in communication and interpersonal skills: the Kalamazoo II report. Acad Med, 2004. 79(6): p. 495–507. doi: 10.1097/00001888-200406000-00002 15165967

[36] Street RL, Buller DB, Patients’ characteristics affecting physician-patient nonverbal communication. Human Communication Research, 1988. 15: p. 60–90.

[37] Li HZ, Krysko M, Desroches NG, Deagle G, Reconceptualizing interruptions in physician-patient interviews: cooperative and intrusive. Commun Med, 2004. 1(2): p. 145–57. doi: 10.1515/come.2004.1.2.145 16808697

[38] DeWane M, Grant-Kels JM, Cell phone use in the clinic: "Please hang up now, the doctor is ready to see you!". Int J Womens Dermatol, 2018. 4(4): p. 238–239. doi: 10.1016/j.ijwd.2017.12.002 30627625 PMC6322151

